# Enhancing recruitment and retention strategies in human tobacco research

**DOI:** 10.1371/journal.pone.0340668

**Published:** 2026-03-12

**Authors:** Laraib Mazhar, Nicolle Krebs, Sophia I. Allen, Andrea L. Hobkirk, Matthew Carrillo, Craig Livelsberger, Vicki Peters, Jonathan Foulds, Jessica M. Yingst

**Affiliations:** 1 Department of Public Health Sciences, Penn State College of Medicine, Hershey, Pennsylvania, United States of America; 2 Department of Psychiatry and Behavioral Health, Penn State College of Medicine, Hershey, Pennsylvania, United States of America; NYU Grossman School of Medicine: New York University School of Medicine, UNITED STATES OF AMERICA

## Abstract

**Introduction:**

Recruitment and retention of study participants in tobacco research studies is challenging, and many studies are not able to meet desired sample sizes, particularly since the COVID-19 pandemic. This survey study explored tobacco users’ motivations and expectations when considering participation in tobacco research and identifies common barriers to participation.

**Methods:**

Participants were adult (21+) tobacco users recruited from a registry of potential research participants. In 2023, participants were emailed a survey to ascertain their motivations for participating in tobacco and health research, their expectations for participation, including compensation and barriers to participating. Means, medians, and proportions were calculated to describe the study population and outcomes. A binary variable for compensation importance was created and analyzed using logistic regression adjusting for demographic and tobacco use factors.

**Results:**

Participants (n = 267) had a mean age of 46.9 years (SD 11.47) and were 67.4% female, 91.0% White, and 3.4% Hispanic. The majority smoked cigarettes (84.3%), while 34.5% reported electronic cigarette use. Most participants (79.4%) indicated interest in tobacco research because they wanted to quit or reduce smoking/tobacco use. Anticipated median compensation for completing a visit lasting one hour with questionnaires was $25, or $50 if a blood draw was included. Two-thirds (66.7%, n = 178) of participants viewed compensation as important. Younger age was associated with the perceived importance of higher compensation (OR=0.971, 95% CI: 0.949–0.993, p = 0.011). The most commonly reported barriers to participation were related to current work schedule issues (41.6%) and not having the ability to take off work when the research center was open (38.6%).

**Conclusion:**

Most participants were interested in tobacco and health research for the intrinsic benefit of quitting or reducing smoking. Removing scheduling and transportation issues with remote research when possible, may increase participation rates. When intrinsic benefit is not available, appropriate compensation should be utilized to boost recruitment and retention rates.

## Introduction

Tobacco use is a public health challenge in the United States, affecting millions of lives each year [1,2]. The United States Food and Drug Administration’s (FDA) Center for Tobacco Products (CTP) is responsible for regulating tobacco products to protect public health. The CTP uses data collected by researchers to inform its regulatory decision-making. Large, randomized, longitudinal studies are considered the gold standard for clinical research [3]. However, recruiting and retaining study participants for these types of trials is challenging [4] and generally, many studies are not able to meet desired sample sizes [5]. Unfortunately, these issues have been exacerbated in the post-COVID-19 era [6].

In order to boost recruitment and retention rates, researchers must understand the motivations to participate in tobacco research, as well as the barriers participants face during a clinical trial. While general barriers to meeting recruitment goals in clinical trials include fear of side effects, time constraints, financial implications, and limited transportation [7,8], barriers specific to tobacco-related research are not well understood. The purpose of this study was to explore tobacco users’ motivations and expectations to participate in tobacco and health studies, and to identify common barriers to participation. These findings can be used to improve recruitment and retention strategies in future tobacco and health research.

## Methods

Participants were adult (21+) tobacco users from a registry of potential research participants maintained by the Penn State Center for Research on Tobacco and Health. Since 2019, this registry has been advertised to potential participants via social media advertisements, community fliers, and medical center communications (fliers, TV screen ads, On-hold phone message). Advertisements included the following example text, “Are you a smoker? Researchers are seeking smokers to participate in various research studies on tobacco and health. To qualify, volunteers must smoke at least five cigarettes per day, be at least 21 years of age, and be able and willing to attend multiple visits in Hershey throughout the study. Compensation for participation and travel is provided.” Those potentially interested in participating in any tobacco-related studies were encouraged to enroll in our participant registry by calling the Center or completing an online screening form. This included providing basic tobacco use information, contact information, as well as consent for future contact. Those entered into the registry were then contacted for studies as they were available. Written consent was waived as the study involved minimal risk and no additional procedures requiring consent. Implied consent was obtained when participants chose to proceed after receiving study information. This procedure, including the waiver of written documentation of consent, was approved by the Penn State University Institutional Review Board.

For this study, all participants in the registry were emailed a link to the survey (n = 1,864). The survey was administered via REDCap electronic data capture tools hosted at Penn State Health Milton S. Hershey Medical Center and Penn State College of Medicine [9]. REDCap (Research Electronic Data Capture) is a secure, web-based application designed to support data capture for research studies. Up to three reminder emails were sent for those who did not complete the survey. At the completion of the survey, participants were emailed a $5 electronic Amazon gift card along with smoking cessation resources.

Demographic questions included biological sex, ethnicity, race, highest educational degree, and US born (yes/no). To assess current tobacco/nicotine use, participants were asked, “Do you currently use cigarettes, electronic cigarettes/vape pens, cigars, pipes, snus/snuff/dip, chew, hookah/waterpipe, or dissolvables, heat-not-burn cigarettes? (yes/no)”. Those indicating yes were asked to report which products were used and how many times per day it was used.

Motivation for participating in tobacco and health research was assessed with the following question, “Please select all the reasons that you were interested in helping with tobacco-related research (to quit smoking/tobacco use, to reduce smoking/tobacco use, to earn compensation or payment for participation, to contribute to research and science, to learn more about my health, other).” For analysis, those reporting interest in quitting or reducing were combined. Participants were also asked about expectations for participation including how far they would be willing to travel to participate in research, how many times they would be willing to return to the study center, communication preferences, and anticipated compensation. For example, participants were asked, “In your opinion, how important is compensation (payment) for participation in research studies? (not at all important, slightly important, important, fairly important, very important), and “How much compensation would you expect for study visit lasting one hour that required you to complete questionnaires, complete basic biological measurements, and give a blood sample?”. A binary variable was created by grouping responses on compensation importance into “important” (important, fairly important, very important) and “not important” (not at all important, slightly important) for logistic regression analysis. Finally, participants were asked about perceived barriers to participating. Participants were asked, “In your opinion, what are the barriers to participating in research studies at Penn State? (no transportation, distance to park and walk, not having trust in the research community, not having trust in the medical community, current work schedule, not having ability to take off during the day when the research center is open, and not enough compensation).”

Statistical analysis was performed using SAS 9.4 statistical package. Means with standard deviations (SD), medians, and proportions were calculated to describe the characteristics of the study population and their experiences. Logistic regression was used to identify variables associated with the outcomes of interest. Age, sex, race, ethnicity, education, US nativity, ever participated in a study, cigarette status, and electronic cigarette status were included in the model as independent variables.

## Results

Participants (n = 267) had a mean age of 46.9 years (SD 11.47) and were 67.4% female, 91.0% White, 3.4% Hispanic, and 97.0% US-born. About 20% obtained Bachelor’s degree or higher. About one-third (33.3%, 89/267) reported ever participating in a study with the Penn State Center for Research on Tobacco and Health. The most used tobacco product was cigarettes, with 84.3% smoking an average of 14.4 cigarettes per day (SD 8.4). About one-third (34.5%) reported current use of electronic cigarettes.

Most participants (79.4%) indicated interest in participating in research because they wanted to quit or reduce smoking/tobacco use. Those who wanted to quit or reduce their smoking were more likely to be female (OR=.52, 95% CI.28-.95). About 40% (106/267) reported interest in participating to earn compensation or payment. Those who were more likely to participate to earn compensation were younger (OR=.96, 95% CI.94-.98) and more educated (OR=1.15, 95% CI 1.03–2.03). Other reasons for participating included to contribute to research and science (67.0%) or to learn more about their health (27.3%).

The most preferred method of communication for contact was email (86.1%) followed by text messaging (56.9%), phone call (21.7%), mail (12.7%), and social media (Facebook messenger) (4.9%). The majority of participants were willing to travel up to 20 miles to participate in research (57.7%) and were willing to return to the study center as many times as needed (64.4%). Only 2.6% of participants indicated that they were not willing to travel (implying they would only participate remotely).

**Fig 1:**
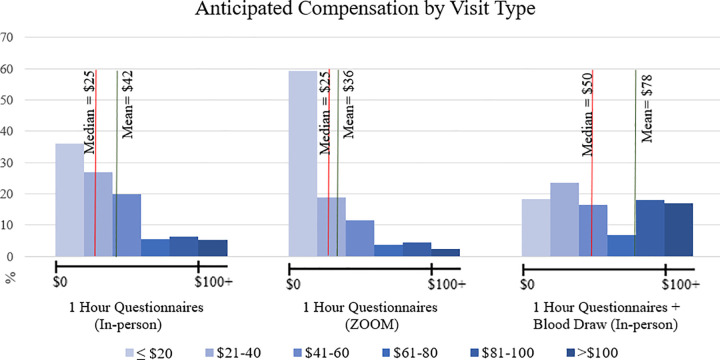
Anticipated compensation by visit type. The anticipated participant compensation is distributed across three visit formats: 1-hour in-person questionnaires, 1-hour Zoom questionnaires, and 1-hour in-person questionnaires with a blood draw, with bars representing the proportion of respondents selecting each category.

Overall, expected median compensation for completing a visit lasting one hour with questionnaires was $25 (Mean = 41.7, SD = 36.9). Expected compensation for completing a visit lasting one hour requiring basic vital measurements and a blood sample was $50 (Mean = 78.4, SD = 82.8) and expected median compensation for a one hour visit completed remotely via phone or ZOOM was $25 (Mean = 35.9, SD = 39.1) (Fig 1).

Overall, about two-thirds of participants (66.7%; 178/267) considered compensation to be important. Among those who thought compensation was important (66.7% *n* = 177), the median anticipated compensation was $40 (Mean = 49.2, SD = 38.6), $75 (Mean = 92.9, SD = 87.0), and $30 (Mean = 44.5, SD = 44.1) respectively. Younger individuals were more likely to report compensation as important, compared to older individuals. For each additional year of age, the odds of finding compensation important decrease by 2.9% (odds ratio = 0.97, 95% CI: 0.949 to 0.993, p = 0.011) adjusting for sex, race, ethnicity, education, US nativity, ever participated in a study, cigarette status, and electronic cigarette status. Since age was the only demographic factor significantly associated with perceptions of compensation importance, further interaction analyses were not conducted.

The most commonly reported barriers to participation were related to scheduling issues including current work schedule (41.6%) and not having the ability to take off work when the research center was open (38.6%). Participants also reported that there was not enough compensation (28.5%), they did not have transportation (26.2%), or the distance to park and walk was too far (20.6%). Only a small proportion reported a lack of trust in the research (3.4%) and medical (4.1%) communities as barriers to participation.

## Discussion

We found that most participants in our study were interested in tobacco and health research as a way to help them quit or reduce their smoking, despite general advertisements that did not mention quitting smoking. This is supported by previous research that suggests most people participate in research for personal benefit, including therapeutic benefits such as quitting smoking [10]. Given that most participants interested in tobacco research want to quit or reduce their smoking, studies without a focus on quitting must consider ways to maximize other potential motivations for participation. In addition, these studies could consider providing cessation support at the conclusion of the study to provide benefits to participants.

Many participants also reported that compensation was a motivating factor for participation, with the younger participants more likely to find compensation important. Participants expected around $50 in compensation for completing a one hour visit including questionnaires and a blood sample. Those who thought compensation was important expected a median of $75. These findings suggest that participants expect compensation rates much higher than minimum hourly wage (which is currently $7.25/hour in Pennsylvania) [11]. However, compensation must be balanced to ensure fair compensation without providing an amount that could be viewed as coercive, particularly with this population, since many people who smoke are of lower socioeconomic status [12]. Cost-related concerns among individuals with lower income were especially influential in deciding to participate in a clinical trial [13]. Our finding that younger individuals viewed compensation as important aligns with and extends the limited literature on demographic predictors of the perceived importance of compensation in tobacco studies. Previous studies found that younger individuals were more motivated to participate in clinical trials when incentives were offered, compared to older adults [14,15]. Other studies have also noted that reasons for participation, such as contributing to research or altruism, were more commonly reported among younger, White participants and those with at least a high school education [16,17]. However, the age-specific effects of compensation on study enrollment and retention remain inconsistent, highlighting the need for further research to identify which demographic subgroups derive the greatest motivation from financial incentives.

While previous research has examined general motivations for clinical research participation, tobacco-specific motivations and barriers remain less well characterized, particularly in studies not focused on cessation. Historically, tobacco studies primarily attracted individuals seeking help to quit, offering intrinsic health benefits. As tobacco regulatory science has expanded to include research studies without a cessation component, understanding participation drivers when intrinsic benefits are minimal is very important. Our findings indicate that monetary compensation plays a significant role in these contexts, aligning with prior research showing that financial incentives are a key facilitator of participation across behavioral and clinical studies, particularly when no direct health benefits were offered [18,19]. Compensation has also been used successfully to promote health behaviors, including smoking cessation among adolescents, substance users, patients with pulmonary disease or serious mental illness, and pregnant women, with larger or escalating payments generally proving more effective than smaller ones [20]. Although compensation is commonly reported as important, most studies also note that altruism, scientific interest, and personal relevance contribute to participation [16,21]. Our survey asked questions about factors relevant to both enrollment and retention, including motivations for participation, scheduling flexibility, transportation, and compensation. Identified barriers and facilitators can influence not only initial participation in tobacco studies but also sustained engagement in studies with longer follow-up periods.

Our study contributes novel insights by quantifying compensation expectations among tobacco users and identifying post-COVID-19 barriers to participation, including scheduling and transportation. Despite greater flexibility in work arrangements since the pandemic [22], participants still reported difficulty attending visits during work hours. This likely reflects our population’s limited job flexibility as well as shifting cultural expectations regarding in-person commitments. These findings are consistent with previous research highlighting persistent barriers such as transportation, parking, childcare, work-hour conflicts, and inadequate trial information [23]. While these issues are common across all types of clinical research [24], a shift towards more remote research may help to alleviate these issues [25]. However, full remote participation is often not possible in tobacco research. The in-person biological verification methods, such as exhaled carbon monoxide (CO) breath tests or biomarker collection (e.g., cotinine or NNAL), often have strict time or temperature constraints, require trained personnel, and raise concerns about reliability if self-administered at home. Conducting remote research for studies on tobacco products is also challenging, as participants must obtain products from researchers, a task complicated by the regulations that prohibit the mailing of tobacco products. In this regard, our findings support the need for hybrid models, such as evening or weekend visits, mobile data collection, or partial remote participation, that can accommodate participants’ schedules while meeting the in-person requirements unique to tobacco research.

Limitations of this study include the small convenience sample collected in central Pennsylvania, which may limit the generalizability of the findings to other geographical regions that may have higher or lower incomes and living costs. The response rate was low (14.32%), and the sample was not representative of the broader population of people who smoke, with most respondents being female, White, and from a single US state. These characteristics likely reflect the composition of our tobacco user registry but may not capture the perspectives of more diverse tobacco user populations. Additionally, we did not ask participants about their income, which prevented us from examining the potential associations between income and expectations for compensation. Another limitation is the exclusive use of an online survey with implied consent, which may have led to potential selection bias, by underrepresenting individuals without internet access or low digital literacy, as well as potential underreporting due to the sensitive nature of tobacco use. Future studies should include larger, more diverse samples and collect detailed sociodemographic data to enhance generalizability and allow for more nuanced analyses.

## Conclusion

Our study findings revealed that most participants were interested in tobacco and health research for the intrinsic benefit of possibly receiving help in quitting or reducing smoking. Removing scheduling and transportation issues with remote research, when possible, may increase participation rates. When intrinsic benefit is not present and the study requires clinic visits during times potentially inconvenient for the participant, appropriate compensation should be utilized to boost recruitment and retention rates.
